# Waterbird counts on large water bodies: comparing ground and aerial methods during different ice conditions

**DOI:** 10.7717/peerj.5195

**Published:** 2018-07-17

**Authors:** Dominik Marchowski, Łukasz Jankowiak, Łukasz Ławicki, Dariusz Wysocki

**Affiliations:** 1 Ornithological Station, Museum and Institute of Zoology, Polish Academy of Sciences, Gdańsk, Poland; 2 Department of Vertebrate Zoology and Anthropology, Faculty of Biology, Szczecin University, Szczecin, Poland; 3 West Pomeranian Nature Society, Szczecin, Poland

**Keywords:** Wintering, Costal lagoons, Baltic Sea, Ducks, Waterfowl, Accuracy of population estimates

## Abstract

The aerial and ground methods of counting birds in a coastal area during different ice conditions were compared. Ice coverage of water was an important factor affecting the results of the two methods. When the water was ice-free, more birds were counted from the ground, whereas during ice conditions, higher numbers were obtained from the air. The first group of waterbirds with the smallest difference between the two methods (average 6%) contained seven species: Mute Swan *Cygnus olor*, Whooper Swan *Cygnus cygnus*, Greater Scaup *Aythya marila*, Tufted Duck *Aythya fuligula*, Common Goldeneye *Bucephala clangula*, Smew *Mergellus albellus* and Goosander *Mergus merganser*; these were treated as the core group. The second group with a moderate difference (average 20%) included another six species: Mallard *Anas platyrhynchos*, Eurasian Wigeon *Mareca penelope*, Common Pochard *Aythya ferina*, Great Crested Grebe *Podiceps cristatus* and Eurasian Coot *Fulica atra*. The third group with a large difference (average 85%) included five species, all of the Anatini tribe: Gadwall *Mareca strepera*, Northern Pintail *Anas acuta*, Northern Shoveler *Spatula clypeata*, Eurasian Teal *Anas crecca* and Garganey *Spatula querquedula*. During ice conditions, smaller numbers of most species were counted from the ground. The exception here was Mallard, more of which were counted from the ground, but the difference between two methods was relatively small in this species (7.5%). Under ice-free conditions, both methods can be used interchangeably for the most numerous birds occupying open water (core group) without any significant impact on the results. When water areas are frozen over, air counts are preferable as the results are more reliable. The cost analysis shows that a survey carried out by volunteer observers (reimbursement of travel expenses only) from the land is 58% cheaper, but if the observers are paid, then an aerial survey is 40% more economical.

## Introduction

Waterbirds are well-known indicators of the quality of aquatic environments. If a particular site holds 1% or more of the flyway population of a given species, this area is said to be important for this population. A flyway is a flight path used in bird migration (Boere & Stroud, 2006) and a flyway population is the number of individuals of a certain species included in a given flyway area. The 1% criterion is used to qualify an area as a wetland of international importance under the Ramsar Convention on Wetlands and by the European Union to identify Special Protection Areas (SPAs) under the Birds Directive. It is also used by BirdLife International for identifying Important Bird Areas (IBAs) on wetlands worldwide (BirdLife International, 2004; BirdLife International, 2015; Wetlands International, 2010). Counting waterbirds on large, open water areas, like marine areas, coastal lagoons and large lakes, is challenging, but accurate counts are critical for estimating population sizes. Different methods have been used to conduct censuses of birds in these open-water environments. Depending on local conditions, there are three main census methods: counting from the ground, aircraft or boats (Komdeur, Bertelsen & Cracknell, 1992; Wetlands International, 2010). The results of censuses carried out by different methods are widely used in species population estimates over larger areas like the Baltic Sea (e.g., Skov et al., 2011; Aunins et al., 2013) or for the whole flyway population of species (Wetlands International, 2018). They are the basis for determining trends in species’ abundances, which in turn affect conservation activities (e.g., Jensen, Perennou & Lutz, 2009). Some studies made the assumption that aircraft counts detected 85% of birds (Johnson, Pollock & Montalbano, 1989). The accuracies of different field protocols for counting birds were tested at different locations (e.g., Briggs, Tyler & Lewis, 1985; Smith, 1995; Frederick et al., 1996; Kingsford, 1999; Frederick et al., 2003; Green et al., 2008). Some papers on non-breeding populations compared the results of air and ground counts in Australia (Kingsford & Porter, 2009), on tidal sea coasts on the Wadden Sea in Denmark (Laursen, Frikke & Kahlet, 2008) and Germany (Scheiffarth & Becker, 2008), and in the Poyang Basin in China (Fawen, Changhao & Hongxing, 2011), but they did not take ice coverage into account. This is a particularly important factor, considering that a large proportion of waterbird species overwinter in areas around the mid-winter 0 °C isotherm (Van Erden & De Leeuw, 2010). Thus, relatively small variations in temperature significantly affect the conditions in which counting is undertaken. Like many other important Baltic bird wintering sites, our study area lies on the mid-winter 0 °C isotherm. The Baltic Sea as a whole is the most important wintering area for waterfowl anywhere in the Western Palearctic (Durinck et al., 1996). Although birds originating from breeding grounds situated in the vast expanses of northern Europe and Asia congregate on the Baltic Sea during the winter, they are not evenly spread: there are few or no birds at all in some areas, but huge numbers of them in others (Skov et al., 2011). These latter ‘hot spots’ are in shallows on the open sea or in the estuaries of rivers where food, mainly mussels and fish, is plentiful (Ławicki, Guentzel & Wysocki, 2012; Marchowski et al., 2015). It happens that a significant percentage of the entire flyway population of a species gathers in a few such optimal places: for example, 14% of the entire Greater Scaup *Aythya marila* population regularly overwinters in the Odra estuary (Marchowski et al., 2017). In the context of climate warming and the related northward and eastward shifts in the wintering range of waterbirds (Lehikoinen et al., 2013; Marchowski et al., 2017), the importance of the Baltic Sea as a wintering area for this group of birds is now far greater than just a few decades ago (Skov et al., 2011).

The investigation of such dynamic ecological processes requires precise research methods. Here, we compare two standard methods of counting birds (from an aircraft and from the ground) under different weather conditions in parts of the south-western Baltic Sea where very large numbers of waterbirds congregate. The specific aim is to test the accuracy of air counts vs. ground counts of waterbirds. Our study area is a key staging and wintering site for significant numbers of a few species of waterbirds from the NW Europe—W Siberia flyway, principally Greater Scaup *Aythya marila*, Smew *Mergellus albellus* and Goosander *Mergus merganser*. Other species, such as Common Pochard *Aythya ferina*, Tufted Duck *Aythya fuligula*, Common Goldeneye *Bucephala clangula*, Eurasian Coot *Fulica atra* and Great Crested Grebe *Podiceps cristatus* are also present in significant numbers (Ławicki et al., 2008; Marchowski & Ławicki, 2011; Marchowski & Ławicki, 2012; Marchowski, Ławicki & Guentzel, 2013).

Although methodological publications mention the high cost of aerial surveys (e.g., Wetlands International, 2010; Meissner, 2011), they do not make any specific calculations. Very few analyses compare the cost of air and ground counts; those that have been performed concern other geographical regions and are out of date (e.g., Kingsford, 1999). Bird counts used for large-scale population estimates often rely on the work of volunteers (Wetlands International, 2010). This significantly reduces costs, which are limited to the reimbursement of travel costs to the surveyed area. In this article we carry out a cost-benefit comparison of the count method (air, ground) and the payment method (volunteering, paid service). This analysis relates to Poland: the financial outlay in other countries will obviously vary, depending on local labour and fuel costs, but the proportions may well be similar and thus more universal.

We pose the following research questions: (1) which of the tested methods gives higher/lower results of counts, and does this depend on ice cover and species? (2) Which method is the more effective and methodologically correct in the context of the financial outlay and ice conditions? Our hypotheses are that: (1) the overall result of a bird count in ice-free conditions is higher from the ground than from the air; (2) the number of birds detected during counts during ice conditions is higher from the air than from the ground; (3) the difference in the counts between the two methods is greater during conditions when ice is present; (4) some species are more sensitive to different census methodologies than others; (5) there are differences in the quality of species identification depending on the method (some species groups are better identified from the ground, others from the air).

## Material and Methods

### Study area

**Figure 1:**
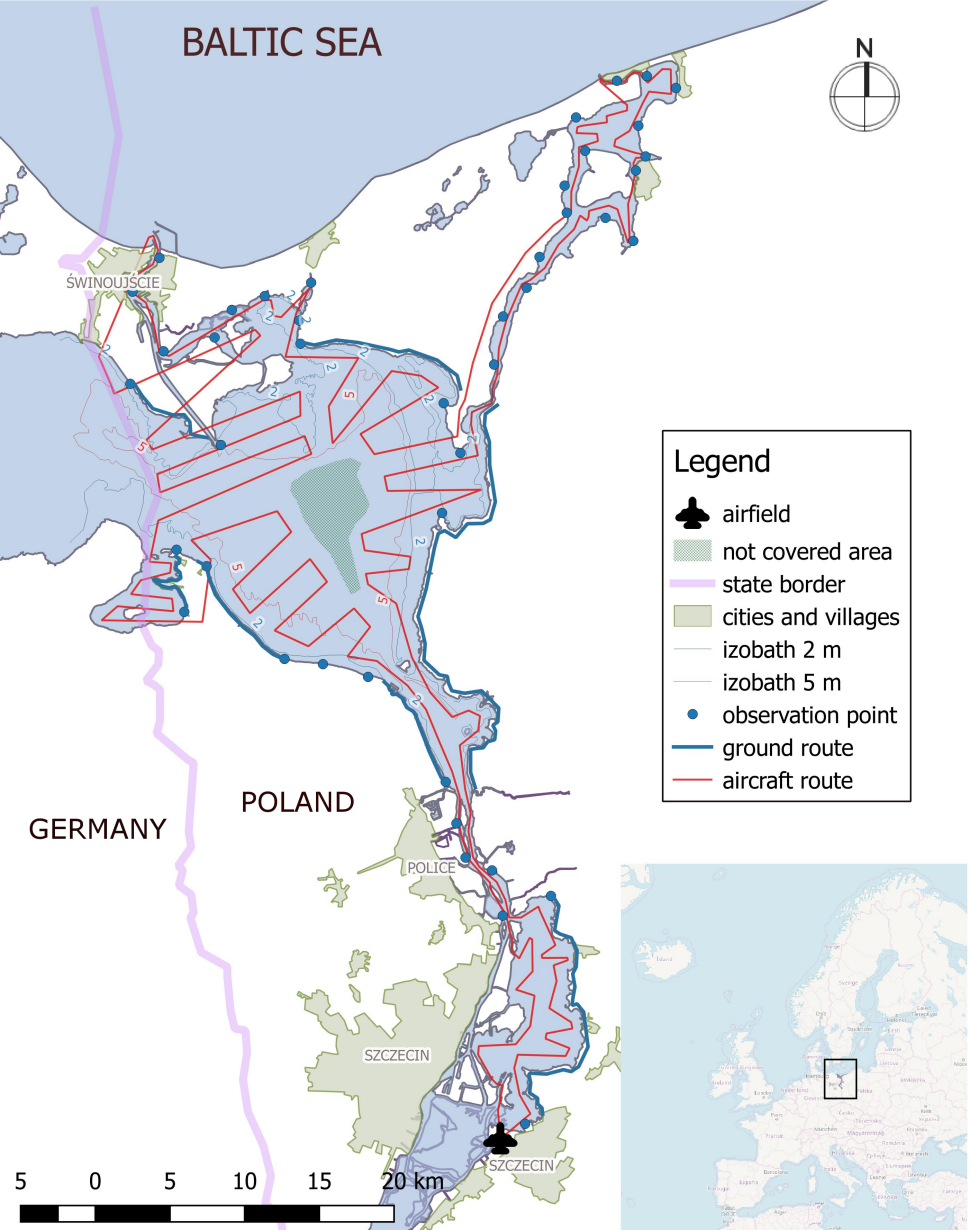
The study area—the Odra River Estuary, NW Poland.

The study area lies in the south-western part of the Baltic Sea and forms the Polish part of the Odra River Estuary system. It covers a total area of 530 km^2^ and includes the Great Lagoon (the Polish part of the Szczecin Lagoon), Świna Backward Delta, Kamień Lagoon, Dziwna Strait and Lake Dąbie (Fig. 1). The average and maximum depths of the Lagoon are 3.8 and 8.5 m, respectively (the dredged shipping lane cutting across the Lagoon from Baltic Sea to the port of Szczecin is 10.5 m deep). The waters of the Szczecin Lagoon, Kamień Lagoon and Lake Dąbie are brackish. The salinity in the central part of the Lagoon varies from 0.3 psu to 4.5 psu (mean = 1.4 psu) and declines with increasing distance from the sea. Periodic inflows of water from the Pomeranian Bay (salinity ∼7 psu) take place through the Świna Strait and, to a lesser extent, through the Dziwna and Peene Straits (the latter in the German part of the estuary). The Odra estuary is subject to strong anthropogenic pressure, which is manifested by a high level of eutrophication (Radziejewska & Schernewski, 2008).

### Counts

We conducted ten aerial counts in parallel with ten ground counts in the non-breeding period during 2009–2014 (see Table S3 for the raw data). Here we consider the following taxa: Great Crested Grebe, Eurasian Coot and Anatidae (ducks, geese and swans). For each taxon there were 10 aerial counts and 10 counts on the ground (in the case of Great Crested Grebe only seven aerial and seven ground counts took place; a total of 394 records for all species). The nomenclature of species and the systematic order used in the article are according to the latest version of the HBW and BirdLife Taxonomic Checklist (Handbook of the Birds of the World (HBW)/Birdlife International Working Group, 2017). The following way of listing species in the text (e.g., Marchowski et al., 2015; Marchowski et al., 2017), standard in ornithological publications, has been adopted: when the species name appears for the first time, the full English name is given along with the full scientific name (genus and species—in italics); whenever a species is referred to again in the text, the English name only is used.

No observations were made during extreme weather conditions (heavy rain, wind, strong wave action). When referring to the ‘census method’ we use the alternative terms ‘platform’ and ‘count method’. All count results were raw data: numbers were not processed by any calculations, such as distance analysis. We used ‘total count’ methods with both platforms and compared the results obtained with both. Air and ground counts took place on the same day. This ‘total count’ method was also used in other studies (Joasen, 1968; Savard, 1982; Kingsford, 1999; Voslamber & Van Turnhout, 1999; Laursen, Frikke & Kahlet, 2008). The same team of 17 trained and experienced observers was involved in all the counts. The research involved observing birds from a distance so as not to disturb them. In Poland, such studies do not require a special permit, as the whole area where we conducted the survey is freely accessible to the public.

A slow-flying, high-wing aeroplane was used for the aerial counts. Two observers identified and counted birds on both sides of the aircraft. The average flight speed was about 100 km/h and the average flying height was about 80 m above the water. This gave a roughly 1,500 m wide band within which birds could be recorded. The altitude of the aircraft flight was tested before the study so that birds could be identified and at the same time not scared off the water. During the study, however, there were a few cases when the birds took off from the water surface at the sight of the aircraft. However, the frightened birds flew off just a small distance away, so they could be tracked from the aircraft and not counted twice. The flight route was designed to cover as much of the water surface as possible; we estimated that coverage was thus approximately 95% of the area surveyed. Only the birds in a very small part of the middle of the Szczecin Lagoon (the largest water body in the survey area—see Fig. 1—‘not covered area’) were not counted: we knew from previous field experience that birds rarely used that area, if at all. The flight route is shown on Fig. 1: the aircraft took off from the Szczecin Aeroklub airfield in Szczecin Dąbie, then flew over Lake Dąbie, the Szczecin Lagoon, Kamień Pomorski and Świnoujście. We used the same flight route for all the aerial surveys. The detailed methodology is adopted from Komdeur, Bertelsen & Cracknell (1992).

Ground counts were usually done on foot, although cars were also involved. Each observer was equipped with 10 ×  40 or 10 × 50 binoculars and tripod-mounted spotting scopes with variable magnification, usually 20–60×. During the counts, observers walked along the same routes, stopping every few hundred metres to scan the area with binoculars and/or spotting scope and then count the birds. Alternatively, counts were conducted from vantage points accessible by car (Fig. 1). We used the best vantage points and routes, dividing the study area up into areas that were visible from such points or routes so that no counted areas overlapped and no parts of the study area were overlooked. Of course, some birds hidden in the rushes may not be detected, which results in an underestimation of the result. On the other hand, it was still possible that the same birds were counted twice by two observers, especially at the points of contact between adjoining sections, at places where the shoreline is strongly indented, or when the same flock of birds swimming far from the shore was being counted by two observers from two different points. Being aware of this problem, we devised methods to reduce the risk of double counting. The observers are in touch by phone and report to each other the species composition and number of birds present at the intersections; they then decide who will include this group to his/her section. In order to eliminate the double counting of flocks of birds away from the shore, we use maps. The observer draws on the map the approximate area and place occupied by the flock. If the other observer marks the flock in a similar place, we only add one number to the result. Which result we add to the general results depends on several factors: which observer saw the flock from a closer distance, the observer’s position in relation to the sun, his/her position in relation to the structure of the landscape, etc. Birds on the water and birds flying (along with the flight direction) are recorded separately. The present analysis took only birds on the water into consideration. All the counts were carried out from the same routes and observation points. The methodology of the ground counts is consistent with generally accepted standards in this field (Komdeur, Bertelsen & Cracknell, 1992; Wetlands International, 2010). The two count methods cannot be compared to a completely unbiased estimator, only with each other.

### Species identification

To compare the effectiveness of the method in species identification, the number of unidentified individuals (identified to generic level only) was compared. For the calculations we used the following groups of birds: (1) Anatini (*Anas sp., Spatula sp., Mareca sp*.) (2) *Aythya sp.* and (3) *Cygnus sp.* Then, the numbers of these groups were compared and the statistical significances of these differences evaluated (see the ‘Statistical analysis’ below).

### Cost accounting

In the case of aircraft counts where the observers are volunteers, only the cost of hiring the aircraft is given. The calculation of volunteer labour costs involves multiplying the rate per kilometre by the number of kilometres that the observer covered, route to the counting site and during the counting. The rate per kilometre covers the costs of fuel and vehicle wear and tear, and is standard in Poland: these rates are set out in the Regulation of the Minister of Infrastructure of 25 March 2002 on the conditions for determining the means of reimbursing the costs of using, for business purposes, cars, motorcycles and mopeds not owned by the employer (Dz.U. 2002 nr 27 poz. 271 available at: http://prawo.sejm.gov.pl/isap.nsf/DocDetails.xsp?id=WDU20020270271). The total number of kilometres driven by all observers using cars during counting (the average of 10 counts) was used in the calculations. The calculation of paid services covers the reimbursement of fuel costs, as well as those of vehicle wear and tear and an expert’s work in Poland (28 €/h). The calculation of the commercial aircraft service covers the costs of hiring the aircraft and the remuneration for highly qualified specialists (43 €/h). The authors and observers are familiar with the hourly rates for the work from their own experience. The costs in Polish zlotys (PLN) were converted into Euro at the average exchange rate on 26 February 2018, i.e., 1 € = 4.2 PLN.

### Statistical analysis

The descriptive statistics were performed using R software (R Development Core Team, 2014) using simple bootstrap to estimate means, standard error and confidence intervals. To check the statistical significance of the differences between groups of unidentified bird species (identified only to the genus level) we used a permutation test based on resampling without replacement (see Script S1).

Waterbirds are characterized by different features such as shape, colour and behaviour, which determine their detectability during counting. Accordingly, we divided the birds into three groups:

Group 1—“core group”: large birds, readily visible on the water from a large distance (swans: Mute Swan *Cygnus olor*, Whooper Swan *Cygnus cygnus*); medium-sized species that usually form large flocks, numerous in the study area, occurring on the open water (Greater Scaup, Tufted Duck, Smew *Mergellus albellus*, Goosander *Mergus merganser* and Common Goldeneye *Bucephala clangula*).

Group 2—medium-sized birds, fairly numerous in the study area, forming large and medium-sized flocks, usually occurring close to the coast and aquatic vegetation, where they can hide (Mallard, Eurasian Wigeon, Common Pochard and Eurasian Coot) or on the open water, well away from coastal vegetation, singly or in small groups (Great Crested Grebe).

Group 3—medium-sized, fairly or not numerous species, forming small flocks or occurring singly, close to the coastal vegetation zone, where they can hide (Gadwall, Eurasian Teal, Northern Shoveler, Garganey).

We used the generalized linear mixed-effect model (GLMM) to analyse the relationship between the results of the aerial and ground counts. To account for the paired nature of the counts (i.e., ground and aerial counts were done on the same day), we added the count date as a random effect; this therefore factors in within-day variation. We also added species as a random factor to account for between-species variation. The number of birds of a target species was the dependent variable. The occurrence of ice (1: over 70% of the water surface covered by ice, 0: no ice observed—see Table S3 for details), count type (Aircraft/Ground) and group of species (levels: Group 1, Group 2, Group 3) were treated as categorical fixed effects. To check how the different counts of particular species and the changes in these numbers were affected by the two counting methods and the presence of ice in relation to the different groups of species, we applied the three-way fixed effect interaction ice*method*group. Because of the high overdispersion of the dependent variable, we used the negative binomial distribution with log-link function. The mixed model was fitted using maximum likelihood. The statistics were performed using R software (R Development Core Team, 2014) with the “lme4” package (Bates et al., 2015). The results were considered statistically significant for *P* < 0.05 and marginally significant at *P* < 0.06.

## Results

### All birds together

There were more birds during ice-free conditions (all species combined and the aggregate number of all individuals, Table 1). The numbers obtained from ground counts in such conditions were higher than from aerial ones (10%, see Table 1). During ice conditions, the overall number of birds was lower than when the water was free of ice, and the difference between the two census methods was much higher (50%, see Table 1).

**Table 1:** Mean number of waterbirds during the non-breeding period in the Odra River Estuary (NW Poland); standard error and confidence intervals, taking into account the method and weather conditions (ice  =0 − no ice, ice  =1 − ice cover over 70%).

Method	Ice	Mean	Standard error	Confidence intervals 95%	
				Lower limit	Upper limit
Aircraft	0	3,907.064	653.361	2,622.483	5,191.645
	1	1,848.523	480.258	904.283	2,792.764
Land	0	4,323.867	706.905	2,934.012	5,713.722
	1	944.410	341.824	272.346	1,616.475

### Species and groups of species

**Figure 2:**
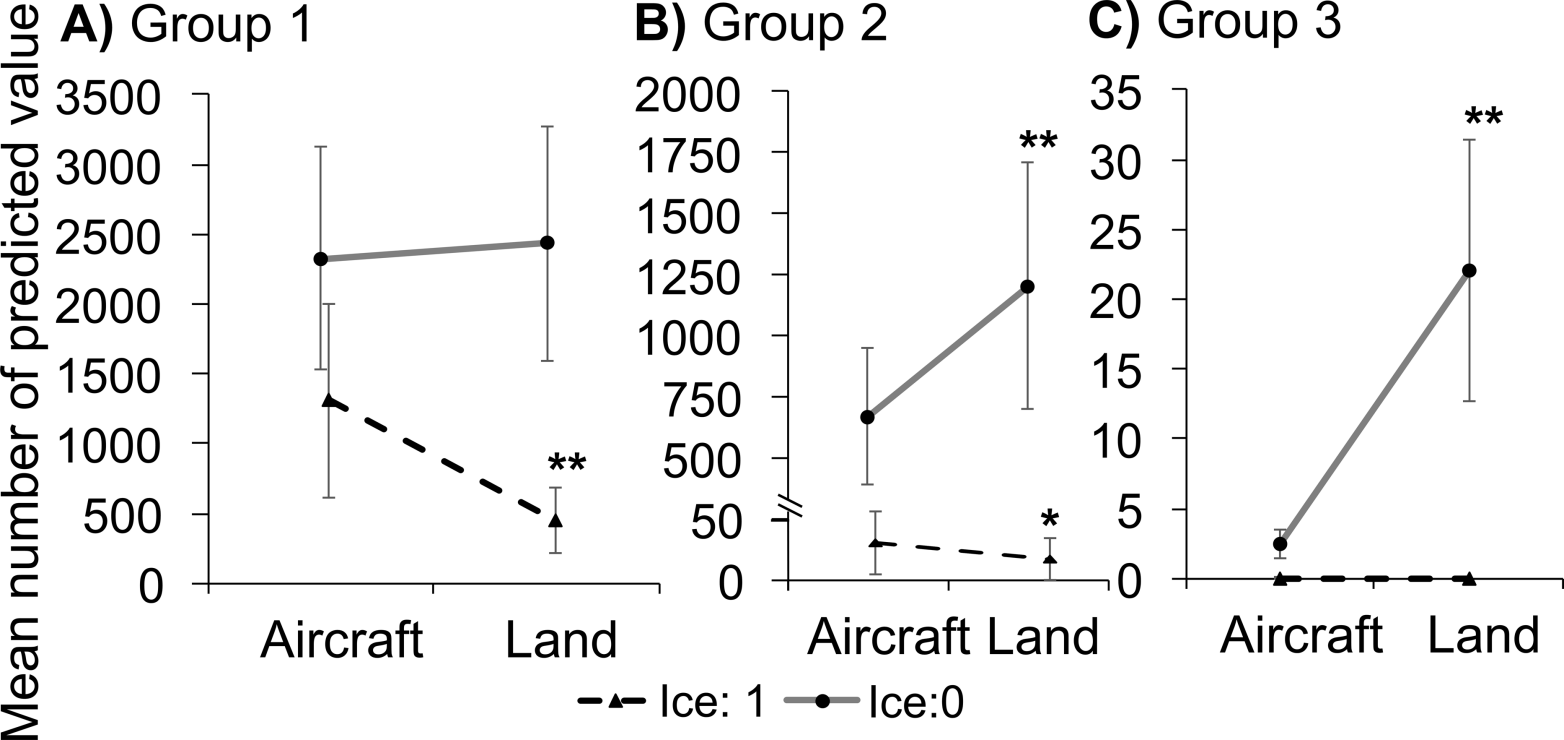
Predicted values of the fitted generalized mixed model. This shows differences between the results of waterbird counts during the non-breeding period in the Odra River Estuary carried out with two research platforms, i.e., from the ground and from the air in relation to different species groups. (A, B, C) show different groups of target species according to different ice conditions and count methods; whiskers indicate standard errors; the asterisk shows a statistically significant difference between Aircraft and Land counts performed as post-hoc tests adjusted by the Tukey method; **, *p* < 0.001; *, *p* < 0.06.

We found a significant three-way interaction between the effect of count method and ice occurrence and groups (Table S2). This indicates that for species from group one (core species) when ice coverage was high, significantly more birds were counted during aerial surveys than ground surveys, whereas when the water was ice-free, the same numbers of birds were counted from the ground and the aircraft (Fig. 2A). For Group 2 species we found that when ice coverage was high, marginally significantly more birds were counted during surveys from the air than from the ground, whereas when the water was ice-free, more birds were counted from the ground (Fig. 2B). In the case of Group 3 species, more birds were counted from the ground when the water was ice-free (Fig. 2C). We did not compare aerial vs ice in relation to Group 3, because when the ice cover was substantial, no birds from this group were counted (Fig. 2D).

The method-related difference during ice conditions was considerable (Table 1), except in the case of Mallard. When waters were free of ice, ground counts were generally higher. In only two cases were the results slightly higher from the air: the differences relating to Greater Scaup and Smew were 0.2% and 1.5% respectively (Table S1). Greater Scaup must be considered in the broader context of the whole *Aythya* genus. A higher air count result is a consequence of the greater efficiency of species identification using this method. Hence, if we consider all the *Aythya* species together, i.e., *Aythya sp*. + *A. marila* + *A. ferina* + *A. fuligula*, the difference is slightly greater (2.8%), but the numbers are still higher from the ground than from the air, as they are for most species (Table S1).

The general range of differences for ice-free waters varied from 0.2% (Greater Scaup) to 93.6% (Northern Pintail) (Table S1).

The group of species with low average levels of difference between the two counting methods (6%) were Mute Swan, Whooper Swan, Greater Scaup, Tufted Duck, Goldeneye, Smew and Goosander—all from Group 1. Species with a moderate average difference level (20%) were Common Pochard, Mallard, Eurasian Wigeon, Great Crested Grebe and Eurasian Coot—Group 2 species. Dabbling ducks (Gadwall, Eurasian Teal, Northern Shoveler, Northern Pintail and Garganey) were the only group with a high level of average difference (85%)—these were species from Group 3.

Under ice conditions, only one species (Mallard) displayed a moderately small difference between the aerial and ground results (7.5%). The other species in such conditions exhibited moderate to high differences—from 34.4% to 582.9%; there was a very wide disparity in differences between species from the same ecological guild (e.g., Tufted Duck—64.6% and Greater Scaup—582.9%; see Fig. 2).

### Species identification

With regard to all recorded birds that could not be identified to species level, there was no statistically significant difference between the methods (*P* = 0.192), although the number of unidentified species was smaller from the aircraft (Table 2).

**Table 2:** Comparison of the number of unidentified species using two counting methods (ground and aircraft) (1); mean ± standard error of ground counts (2); 95% confidence intervals of ground counts (3); mean ± standard error of aircraft counts (4); 95% confidence intervals of aircraft counts (5); *P* value (6).

Genus (1)	Ground		Aircraft		*P* value (6)
	Mean ± SE (2)	95% CI (3)	Mean ± SE (4)	95% CI (5)	
All species	1,194 ± 466.11	409–2,215	517 ± 151.83	256–846	0.192
*Anatini*	80 ± 30.97	26–146	277 ± 56.77	163–385	0.012
*Aythya* sp.	3,392 ± 1,110.03	1,452–5,759	1,075 ± 387.28	387–1,890	0.064
*Cygnus* sp.	111 ± 40.17	38–195	198 ± 85.76	59–387	0.454

Looking at each of the three groups separately, we see important differences between them. The group with the most significant differences is Anatini (*Anas*, *Spatula* and *Mareca*): the number of unidentified birds was significantly higher from the aircraft. In the case of the *Aythya* group, more birds counted on the ground were unidentified (this result is marginally significant, *P* = 0.064). Differences in the number of unidentified *Cygnus* swans were non-statistically significant, although more unidentified swans were observed from the aircraft (Table 2).

### Cost estimate

All the counts for this study were carried out by volunteers; some persons even waived the reimbursement of travel costs to the counting site. The costs involved in this study were low, being limited to the hiring fee for the aircraft and part of the fuel costs for ground observers’ cars. They were even lower than the following calculations in relation to volunteers. However, if we include the fuel cost for all observers and the cost of aircraft hire, we obtain the real overall cost of voluntary counts. Reimbursing the 12 ground observers involved in the counting for their fuel outlay amounts to around 300 € and the aircraft hire fee is 720 €. The study area covers 530 km^2^ and the coastline is 340 km long, so the cost of an air count is 136 € for 100 km^2^ of a water body and 180 € for 100 km of flight route if the count is carried out by volunteers. The corresponding ground count costs are 57 €/100 km^2^ and 88 €/100 km of coastline. If the observers are paid for their services, the costs increase to 1,400 € for an air count and 2,300 € for a ground count (see Table 3 for details). These figures cover only the labour costs in the field and do not include the costs of subsequent data processing and analysis.

**Table 3:** Waterbird counts in the non-breeding season − calculation of costs. Calculation of labour costs in the field; payment methods and study methods are distinguished.

Form of payment	Form of counting	Cost of one count in the study area (530 km^2^ and 340 km of coastline) in Euros	Cost of one count over a 100 km^2^ water body in Euros	Cost of one count along a 100 km coastline (ground count) and 100 km of flight route (air count) in Euros
Voluntary	Aircraft	720	136	180
Voluntary	Ground	300	57	88
Paid service	Aircraft	1,400	264	350
Paid service	Ground	2,300	434	677

## Discussion

The major factor affecting the census results was ice cover. In ice-free conditions, ground counts yielded higher numbers of birds than aerial ones. When ice was present, more birds were counted from the air, and the difference between the two methods was much greater than in ice-free conditions. This discrepancy highlights the importance of ice coverage on the water in impacting survey results in relation to the survey method. With respect to the core species combined, it does not really matter which censusing method is used during ice-free conditions as the counts are not specially affected by this (Fig. 2): the two methods can be used interchangeably. Similar conclusions were reached by Kingsford, Brandis & Porter (2008) in Australia, where the correlation of results from the land and from the air was highly significant. The results of air and ground counts also differed little in the Poyang Basin in China (Fawen, Changhao & Hongxing, 2011).

In contrast, once there is significant ice cover of the waters (above 70%), the survey method does become important; this has not been demonstrated before. Our aerial census results under such conditions gave higher numbers relative to ground counts. During ice conditions, there were a number of occasions when significant numbers of birds were overlooked by ground observers but were recorded from the aircraft on their sections. This is why greater numbers of birds are counted from the air in ice conditions; hence, the aerial method is more effective under such conditions. Wetlands International (2010) recommends the aerial method in areas covered (incompletely) by ice but does not underpin this assertion with any concrete results; our work supports it. Again, in Australia, there are similarities, such as limited visibility from the land on lakes such as Lake Illawarra and Norring Lake, where aerial counts yielded much higher numbers of birds than ground ones (50.1% and 101.5% respectively; Kingsford, Brandis & Porter, 2008). The similarity lies in the limited visibility from the land of sites where significant numbers of birds congregate.

The differences in the results varied over a very wide range—from nearly identical, i.e., 0.2% for Greater Scaup under ice-free conditions, to 582.9%, also for Greater Scaup but in ice conditions. This very considerable difference under ice conditions in the case of Greater Scaup emerges from this species’ preference to concentrate in a few places, i.e., in ice-free areas usually far from the shore (Johnsgard, 1978; Mendel et al., 2008). In ice conditions, several thousand Greater Scaup were recorded from the aircraft in ice-free patches of water not visible to ground observers. Visibility from the land in ice conditions is often difficult because piles of ice protrude above the waterline, a problem that ceases to exist when counting from the air. The much lower difference with regard to the Tufted Duck (sympatric with Greater Scaup) is due to the tendency of this species to occupy anthropogenic sites like ports and harbours when ice covers more open sea areas (Jakubas, 2003), as does Mallard (Meissner et al., 2015); they are thus more easily detected by ground observers. In ice conditions the numbers of most species were higher when counted from the air, Mallard being the exception. We recommend aerial surveys when waters are frozen over. Even if, as seen from the land, the entire water body appears to be frozen, from the air we can still find unfrozen patches, which are occupied by many birds.

The opposite situation prevails when waters are free of ice: ground count numbers are then generally higher. This corresponds with most papers on this topic, in which ice conditions were either not analysed or did not exist (e.g., Pollock & Kendall, 1987; Kingsford, 1999; Laursen, Frikke & Kahlet, 2008). If we take into account particular species of birds, comparable results under ice-free conditions can be obtained by both methods with respect to the following species: Greater Scaup (difference 0.2%), Smew (1.5%), Mute Swan (3.9%), Goosander (4.9%), Common Goldeneye (5.3%) and Tufted Duck (5.5%) (see Table S1). The most numerous species of waterbirds in the study area, they make up the core of the waterbird community here (79–85% of all waterbirds present in the area). The observed difference (6% in core species group) between methods may be acceptable in some situations for the two methods to be used interchangeably. However, researchers must consider the influence these small differences between methods might have on their results and consider developing correction factors where these differences are deemed unacceptable.

In ice-free conditions, comparable results are obtained for numerous birds occupying the open water. We can generalize that diving ducks (*Aythya, Mergus, Mergellus*, *Bucephala*) and swans (*Cygnus*) can be counted from the air without significant differences between the methods. The differences between count numbers are higher for the less numerous birds occupying abundantly vegetated near-shore areas, so we do not recommend surveying these species from the air. Generally speaking, this applies to dabbling ducks (Anatini): here there are significant differences between the methods, with aerial counts being lower relative to ground counts (see Table S1 for more details).

In most cases the ground observer identifies species more accurately than his/her counterpart from the air. The exception of Greater Scaup and Tufted Duck must be explained here. Birds often swim on the water far away from the shore—perhaps as much as a few kilometres. In such a situation, it is difficult to identify the species even with a spotting scope from the shore. In addition, at the level of a single flock, Greater Scaup prefers deeper areas (further from the shore) and Tufted Duck shallower waters (closer to the shore), so an observer from the shore watching such a mixed flock sees Tufted Duck in the foreground and Greater Scaup farther away. As this makes it difficult or even impossible to separate these two species accurately, observers sometimes decide to treat them at the generic level (*Aythya sp*. = Greater Scaup*/*Tufted Duck). The situation is different from an aircraft. Such a flock is seen from a much shorter distance and the spatial distribution is much better visible from the air. Species giving the impression of a mixed flock from the shore, can be seen from the aircraft that they are actually occupying slightly different areas (within one flock), which makes it easier to count them separately.

As we stated in the Methods section, the two count methods cannot be compared to a completely unbiased estimator, only with each other. However, the method yielding higher total counts can be regarded as superior. Such a conclusion is based on experience from the area; for example, one or two species are deliberately overlooked from the air as they can be equally well counted from the ground (during ice-free conditions). On the other hand, during ice conditions, ground observers may not report birds of a given species at all on their sections, whereas significant numbers of this species are observed (a few cases) from the aircraft on these sections. Of course, double counting is still possible: especially at the points of contact between adjoining sections, or where the shoreline is strongly indented, two observers may count the same flock of birds situated far offshore from two different points. Being aware of this problem, we have developed specific methods to reduce this risk (described in Methods).

Any broader application of our results is limited by the fact that our survey covered just one area. But they can be of use more generally in other similar areas in the same climatic zone, where wintering waterbirds congregate. These include the estuaries of large rivers where there are extensive shallow lagoons, such as the German part of the Odra estuary, the Vistula Lagoon, the Curonian Lagoon, or the indented coastline of the south-western Baltic Sea.

The most economical form of counting is to use volunteer observers on the ground: this is the method most commonly used in our study area and generally during winter waterbird counts worldwide (Wetlands International, 2010). The disadvantage of this approach, however, is that one needs a large group of qualified observers equipped with good optical equipment who will not get paid for their services. This condition cannot always be met. Counting from an aircraft requires only two people and, assuming that they will not get paid for their services, the costs are also not high, but still 58% higher than for a ground count. If the observers are paid, then the aircraft method will be the most cost-effective: only two qualified people are needed and the cost is 40% less than for all the persons involved in a ground count (Table 3). In addition, aerial surveys can be used in both ice-free and ice conditions. The present calculation of costs relates to conditions in Poland; in other countries, costs will vary depending on local labour and fuel costs, but the proportions should be similar. The undisputable disadvantage of the aircraft method is its riskiness (Sasse, 2003); in addition, surveys may need to be conducted in places where there are airspace restrictions, stricter limitations regarding weather conditions suitable for counting, difficulties in identifying taxa similar in appearance, and, in some situations, high levels of disturbance to wildlife.

## Conclusions

Overall, more birds are counted from the ground than from the air in ice-free conditions, but in ice conditions, the overall results of bird counts are higher from the air than from the ground. The differences in counts between the two methods are higher in ice conditions. In ice-free conditions, the results from both platforms for the core group of birds occupying open water (diving ducks and swans) are comparable. In the same conditions there are significant differences between the methods regarding two other groups of birds—aerial counts yield lower numbers.

##  Supplemental Information

10.7717/peerj.5195/supp-1 Table S1 Group of waterbird species used to test the accuracy of air and ground counts (1); mean ± standard errors of ground counts (2); 95% confidence intervals of ground counts (3); mean ± standard errors of air counts (4); 95% confidence intervals of air counts (5); method error—difference between mean numbers of birds recorded during ground and air counts (ground minus air) (6); method error—difference between mean numbers of birds recorded during ground and air counts; the value from column 7 is given as the percentage of the mean number of birds obtained from the ground (7)

10.7717/peerj.5195/supp-2 Table S2 Results of general linear mixed models showing (GLMM) the influence of different count methods (Ground vs Aircraft) performed in different ice cover presence (1—ice cover present, 0—no ice cover) in relation to different groups of species (Group 1: Cygnus olor, C. Cygnus, Aythya marila, A. fuligula, Mergellus albellus, Mergus merganser, Bucephala clangula; Group 2: Anas platyrhynchos, Mareca penelope, Aythya ferina, Fulica atra, Podiceps cristatus; Group 3: Mareca strepera, Anas crecca, Spatula clypeata, Anas acuta, Spatula querquedula). Count dates and species were treated as random effects (r) and these are given as a variance with standard error

10.7717/peerj.5195/supp-3 Table S3 Excel spreadsheet of raw data

10.7717/peerj.5195/supp-4 Script S1 Script to R for simple bootstrap to estimate means, se and confidence intervals and permutation test based on resampling without replacement

## Additional Information and Declarations

### Data Availability

The following information was supplied regarding data availability: The raw data are provided in a Supplemental File.
