# Supplementary material for: Waterbird counts on large water bodies: comparing ground and aerial methods during different ice conditions
Source: PeerJ. 2018 Jul 17;6:e5195. doi: 10.7717/peerj.5195 (PMC6054062; doi:10.7717/peerj.5195)
Supplement: Table S1 [file peerj-06-5195-s001.docx]

S1. Table. Group of waterbird species used to test the accuracy of air and ground counts (1); mean ± standard errors of ground counts (2); 95% confidence intervals of ground counts (3); mean ± standard errors of air counts (4); 95% confidence intervals of air counts (5); method error – difference between mean numbers of birds recorded during ground and air counts (ground minus air) (6); method error – difference between mean numbers of birds recorded during ground and air counts; the value from column 7 is given as the percentage of the mean number of birds obtained from the ground (7).

| Species (1) | Ground | | Aircraft | | Meth.error (6) | Method error% (7) |
| --- | --- | --- | --- | --- | --- | --- |
|  | Mean±SE (2) | 95% CI (3) | Mean±SE (4) | 95% CI (5) |  |  |
| *Anas acuta** | 156±101.09 | 23-379 | 10±5.71 | 1-22 | 146 | 93.590 |
| *Anas clypeata** | 51±34.68 | 6-127 | 6±3.67 | 0-14 | 45 | 88.235 |
| *Anas crecca** | 199±57.06 | 105-325 | 47±20.26 | 11-89 | 152 | 76.382 |
| *Anas penelope** | 1011±458.62 | 198-1915 | 690±339.79 | 96-1343 | 321 | 31.751 |
| *Anas platyrhynchos** | 7460±1606.76 | 4505-10777 | 5543±1278.84 | 3200-8200 | 1917 | 25.697 |
| *Anas platyrhynchos*** | 1740±623.55 | 220-2643 | 1609±412.012 | 626-2300 | 131 | 7.529 |
| *Anas querquedula** | 12±6.44 | 1-26 | 0±0 | 0-0 | 12 | 100.000 |
| *Anas strepera** | 75±17.60 | 42-110 | 24±9.10 | 7-42 | 51 | 68.000 |
| *Aythya ferina** | 1157±460.25 | 372-2147 | 636±282.27 | 150-1229 | 521 | 45.030 |
| *Aythya ferina*** | 1±0.54 | 0-2 | 3±2.72 | 0-10 | -2 | -200.000 |
| *Aythya fuligula** | 29932±4088.31 | 22107-38110 | 28282±3733.26 | 20857-35429 | 1650 | 5.512 |
| *Aythya fuligula*** | 3973±813.09 | 2000-5200 | 6540±668.86 | 5200-8030 | -2567 | -64.611 |
| *Aythya marila** | 34368±11488.34 | 17274-59816 | 34426±10873.63 | 17857-58571 | -58 | -0.169 |
| *Aythya marila*** | 917±224.16 | 450-1400 | 6262±1703.58 | 2538-9750 | -5345 | -582.879 |
| *Bucephala clangula** | 3728±969.31 | 2031-5789 | 3532±977.11 | 1877-5654 | 196 | 5.258 |
| *Bucephala clangula*** | 908±549.94 | 0-2223 | 1820±925.47 | 0-3900 | -912 | -100.441 |
| *Cygnus cygnus** | 349±154.36 | 125-693 | 292±104.19 | 121-514 | 57 | 16.332 |
| *Cygnus cygnus*** | 70±29.45 | 20-140 | 236±79.60 | 98-424 | -166 | -237.143 |
| *Cygnus olor** | 546±126.41 | 354-827 | 525±113.13 | 354-827 | 21 | 3.846 |
| *Cygnus olor*** | 180±99.83 | 20-420 | 378±203.00 | 34-861 | -198 | -110.000 |
| *Fulica atra** | 5504±1115.47 | 3623-7555 | 4646±992.21 | 2829-6677 | 858 | 15.589 |
| *Fulica atra*** | 668±319.71 | 235-1450 | 1780±981.61 | 200-4140 | -1112 | -166.467 |
| *Mergellus albellus** | 1324±375.23 | 655-2102 | 1344±453.22 | 570-2324 | -20 | -1.511 |
| *Mergellus albellus*** | 922±472.67 | 200-2067 | 2646±711.28 | 939-3800 | -1724 | -186.985 |
| *Mergus merganser** | 7524±2755.30 | 3123-13608 | 7158±2166.95 | 3239-11686 | 366 | 4.864 |
| *Mergus merganser*** | 7122±3902.16 | 2000-16673 | 12668±4686.70 | 5588-24000 | -5546 | -77.871 |
| *Podiceps cristatus** | 390±139.68 | 159-679 | 244±117.62 | 64-510 | 146 | 37.436 |
| *Podiceps cristatus*** | 32±5.66 | 24-40 | 43±2.12 | 40-46 | -11 | -34.375 |
| *Aythya* all species*** | 50675±13849.07 | 25912-79788 | 49252±12165.43 | 27856-75129 | 1423 | 2.808 |

*no ice counts only (excluded counts witch ice cover over 70%)

**counts with ice cover over 70%

*Anas* duck (except of Mallard *Anas platyrhynchos*) were excluded from calculation during the ice cover condition (**) due to generally lack of this species in such condition.
