# Supplementary material for: Waterbird counts on large water bodies: comparing ground and aerial methods during different ice conditions
Source: PeerJ. 2018 Jul 17;6:e5195. doi: 10.7717/peerj.5195 (PMC6054062; doi:10.7717/peerj.5195)
Supplement: Table S2 [file peerj-06-5195-s002.docx]

S2 Table. Results of general linear mixed models showing (GLMM) the influence of different count methods (Ground vs Aircraft) performed in different ice cover presence (1 – ice cover present, 0 – no ice cover) in relation to different groups of species (Group 1: *Cygnus olor*, *C. Cygnus*, *Aythya marila*, *A. fuligula*, *Mergellus albellus*, *Mergus merganser*, *Bucephala clangula*; Group 2: *Anas platyrhynchos*, *Mareca penelope*, *Aythya ferina*, *Fulica atra*, *Podiceps cristatus*; Group 3: *Mareca strepera*, *Anas crecca*, *Spatula clypeata*, *Anas acuta*, *Spatula querquedula*). Count dates and species were treated as random effects (r) and these are given as a variance with standard error.

|  | Estimate | s.e.. | z value | P |
| --- | --- | --- | --- | --- |
| (Intercept) | 7.763 | 0.652 |  |  |
| Method[Land] | 0.045 | 0.078 | 0.570 | 0.569 |
| Ice[1] | -0.549 | 0.426 | -1.289 | 0.197 |
| Group[Group_2] | -1.311 | 1.024 | -1.280 | 0.200 |
| Group[Group_3] | -6.797 | 1.030 | -6.599 | <0.001 |
| Method[Land]*Ice[1] | -1.111 | 0.149 | -7.461 | <0.001 |
| Method[Land]*Group[Group_2] | 0.543 | 0.126 | 4.309 | <0.001 |
| Method[Land]*Group[Group_3] | 2.147 | 0.160 | 13.412 | <0.001 |
| Ice[1]*Group[Group_2] | -2.363 | 0.690 | -3.423 | 0.001 |
| Ice[1]*Group[Group_3] | -3.165 | 0.943 | -3.355 | 0.001 |
| Method[Land]*Ice[1]*Group[Group_2] | 0.112 | 0.259 | 0.430 | 0.667 |
| Method[Land]*Ice[1]*Group[Group_3] | -3.512 | 1.128 | -3.113 | 0.002 |
| Date of count (r) | 2.535 | 1.592 |  |  |
| Species (r) | 2.717 | 1.648 |  |  |
